# Sleeve Gastrectomy Outcomes in Patients with BMI Between 30 and 35–3 Years of Follow-Up

**DOI:** 10.1007/s11695-017-2897-x

**Published:** 2017-10-04

**Authors:** Marcos A. Berry, Lionel Urrutia, Patricio Lamoza, Alfredo Molina, Eduardo Luna, Federico Parra, María J. Domínguez, Rodrigo Alonso

**Affiliations:** Bariatric and Metabolic Center, Department of Surgery, Clinica Las Condes, Lo Fontecilla 441, Las Condes, Santiago, Chile

**Keywords:** Sleeve gastrectomy, Obesity grade 1, Mild obesity, BMI, Comorbidities, Bariatric surgery

## Abstract

**Introduction and Purpose:**

Laparoscopic sleeve gastrectomy (LSG) in patients with a BMI between 30 and 35 kg/m^2^ plus comorbidities has shown to be safe and effective. The purpose of this study is to describe our outcomes in this group of patients after 3 years of follow-up.

**Materials and Methods:**

Retrospective descriptive analysis of patients with initial BMI between 30 and 35 kg/m^2^ plus comorbidities were submitted to LSG between 2006 and 2013. We analyzed gender, age, comorbidities, BMI, total weight loss (%TWL), excess weight loss (%EWL), comorbidity resolution, morbidity, and mortality. Postoperative success was defined as %TWL over 20% and EWL% over 50% maintained for at least 1 year and comorbidity remission with no need of medication.

**Results:**

Of the patients, 477 underwent a LSG in the above period and 252 met inclusion criteria; 188 (75%) were female and 64 (25%) were male. Median age was 39 years (15–70). Three-year follow-up was 43.9% (111 patients). Median preoperative BMI was 32.3 kg/m^2^ (30–34.3). Median postoperative %TWL was 12.9, 23.2, 28.2, 24.3, and 22.1% at 1, 6, 12, 24, and 36 months, respectively. %EWL was 42.88, 77.44, 98.42, 83.2, and 75.8%. Median surgical time was 86.9 min (40–120). There was comorbidity remission at 36 months. Insulin resistance was remitted in 89.4%, dyslipidemia 52%, non-alcoholic fatty liver disease 84.6%, hypertension 75%, and GERD 65%. T2DM had 60% of complete remission and 40% improvement. There were morbidity in six patients (2.4%), two reoperations, no leaks, and no mortality.

**Conclusions:**

Performing LSG in patients with grade I obesity is safe and effective. BMI should not be the only indicator to consider bariatric and metabolic surgery. We still require further studies and longer follow-up.

## Introduction

Obesity, defined as a body mass index (BMI) >30 kg/m^2^, is a chronic, serious, and costly disease. It is associated with an increased risk of several comorbidities, including type 2 diabetes mellitus (T2DM), cardiovascular diseases (CVDs), and some types of cancer [1]. Obesity’s rate in the world has nearly doubled since 1980s especially in developing countries [2]. The World Health Organization declared obesity as an epidemic disease affecting more than 500 million adults worldwide [3]. Also, the percentage of children with obesity is increasing rapidly especially in low- and middle-income countries, reaching 40 million in 2012 [4].

Class I obesity is the beginning of health deterioration and organ failure as result of progressive fat accumulation [5]. Surgery in mildly obese patients not only seeks to improve esthetic appearance, certainly of great importance because of the psychological and social impact of obesity on people who suffers from it, but also is a way to prevent progression of the long list of comorbidities [6–9].

Different international guidelines suggest that candidates to bariatric and metabolic surgery are only those patients with BMI over 40 (morbid obesity), BMI over 35 (severe obesity), and, at least, two obesity-related comorbidities and inability to achieve a healthy weight loss sustained for at least a year with prior weight loss efforts [10, 11].

Moreover, it has been shown that bariatric surgery is safe and effective in patients with BMI between 30 and 35 kg/m^2^, and international associations currently support this indication [12, 13].

According to Chilean “Comprehensive obesity treatment program” [14], in order to indicate surgery, a multidisciplinary team must evaluate BMI (≥35 with comorbidities or ≥40), comorbidities, food habits, age, patient expectations, adherence to the program, support network, and absence of contraindications. However, exceptions to these indication criteria could be done if the patient’s case is discussed and bariatric surgery is approved and supported by the committee.

Laparoscopic sleeve gastrectomy (LSG) is a restrictive procedure where about 80% of the stomach are removed, producing an extra hormonal regulation that helps patients to improve their eating habits without significant anatomical-functional modification [15–17].

The aim of this study is to describe medium-term outcomes in patients with BMI between 30 and 35 kg/m^2^ submitted to LSG in terms of weight loss and comorbidity improvements over 3 years of postoperative follow-up.

## Patients and Methods

### Study Design and Data Set

Retrospective descriptive analysis of patients was submitted to a sleeve gastrectomy in a specialized center between January 2006 and December 2013 at Clínica Las Condes, Santiago, Chile.

We selected all the patients with a BMI of 30 to 35 and at least one associated comorbidity, undergoing sleeve gastrectomy in the mentioned period. All the patients were assessed by a multidisciplinary team. Informed consent was obtained from all individual participants included in the study or their authorized representative. All the patients below 18 years old were discussed in a specialized committee. Our local ethic committee revised the study.

Comorbidities were studied and followed up with complete blood count, fasting glucose, glycated hemoglobin (HbA1c), insulin, homeostatic model assessment (HOMA), blood urea nitrogen (BUN), creatinine, electrolytes, lipid panel, hepatic panel, thyroid-stimulating hormone (TSH), chest X-ray, electrocardiogram, abdominal ultrasound, and upper gastrointestinal endoscopy (UGE). We also registered number of medication needed to treat these diseases before and after surgery.

### Inclusion Criteria

Patients with BMI between 30 and 35 kg/m^2^ with at least one obesity-related comorbidity such as type two diabetes mellitus (T2DM), insulin resistance (IR) or impaired fasting glucose (IFG), hypertension (HTN), dyslipidemia, and non-alcoholic fatty liver disease (NAFLD) were included. These related diseases were diagnosed and followed up according to the following criteria:

Diabetic patients included had and adequate glycemic control with their medication according to the American Diabetes Association criteria and they were followed up with fasting glucose, glycated hemoglobin (HbA1c), and medication requirement. Patients with IR, impaired fasting glucose, or impaired glucose tolerance (IGT) were diagnosed and followed up with HOMA and oral glucose tolerance test [18, 19]. HTN and dyslipidemia were diagnosed according to the American Heart Association criteria [19–21]. NAFLD was diagnosed after excluding other causes of secondary liver fat accumulation, with abdominal sonography, hepatic profile [22]. No liver biopsy was done for this purpose. Finally, all patients were evaluated with upper gastrointestinal endoscopy, and only those with symptomatic GERD or pathological endoscopy barium esophagogram, esophageal manometry, and esophageal pH test was performed.

Age was between 15 and 70 years old. Adolescents under 18 years old must have both parents consent; skeletal maturity must be clinically demonstrated and mental maturity evaluated by a specialized psychologic team with the whole interdisciplinary committee approval.

### Exclusion Criteria

Excluded were those with insufficient follow-up or insufficient clinical data to evaluate comorbidity evolution after surgery, patients with pregnancy during the follow-up, active uncontrolled psychiatric illness or substance abuse, patients with type I diabetes, and T2DM with more than 10 years of diagnosis, poor glycemic control (HbA1c >7%) even with intensive medical treatment, with oral medication, or insulin. In these severe diabetic patients, with no response to more than two drugs or insulin requirement, a laparoscopic Roux-en-Y gastric by-pass (RYGB) was indicated, as well as in patients with grade C and D esophagitis or Barrett demonstrated by esophagus biopsy.

The selected data was registered in an Excel® database. The variables analyzed were age, sex, comorbidities, medication intake, total weight loss (%TWL), excess of weight loss (%EWL), BMI, and laboratory test results before surgery and subsequently at 1, 6, 12, 24, and 36 months of follow-up. The %TWL was calculated using the following formula: (weight loss/initial weight) × 100. The %EWL was calculated using the formula (weight loss/baseline excess weight) × 100, where excess weight = initial weight − ideal weight (ideal BMI = 23 kg/m^2^).

Postoperative success was defined as %TWL >20 or %EWL over 50% maintained at least for a year. [19, 23] Postoperative comorbidity resolution, remission, or improvement was defined according to the American Society for Metabolic and Bariatric Surgery outcome reporting standards [19].

### Surgical Procedure

Patients were operated with a four-port laparoscopic vertical gastrectomy technique in French position. The sleeve was performed from antrum to the angle of Hiss, starting at 5 cm from pylorus and with a 36 f. boogie calibration. Left hiatal crus was always exposed in order to find and repair any possible hiatal hernia. Reinforcement of the stapler line was made with absorbable buttressing material, and a drainage was placed along the suture line. All the procedures were done by the same team in Clínica Las Condes.

### Statistical Analysis

Data analysis was performed using STATA statistical software (version 12.0; Stata Corp LP, College Station, TX).

Initial biometric data analyzed were age, initial weight, BMI, and EW. We compared median %EWL and BMI at baseline, 1, 6, 12, 24, and 36 months, respectively. Categorical variables are expressed as number of cases (*n*) and percentages. The normal distribution continuous variables were assessed with the Shapiro-Wilk test. Non-parametric variables were expressed in medians and interquartile range (IQR). Wilcoxon signed rank test was used to compare changes in continuous variables over time. The previous presence of comorbidities and its resolution after surgery was compared and analyzed with Fisher exact test. A *p* value <0.05 was defined as statistical significance.

## Results

### Initial Biometric Data

From 477 patients who underwent LSG, a total of 225 patients were excluded because of insufficient clinical data to evaluate comorbidity evolution. Percentage of follow-up was 82.6 (209 patients) the first month, 88.1% (223 patients) at 6 months, 87% (220 patients) at 1 year, 50.6% at 2 years (128 patients), and 43.9% (111 patients) at 3 years. Of the 252 patients selected, 188 (75%) were women and 64 (25%) men (Table 1). The median age was 39 years old. They had an initial median weight of 86 kg, BMI of 32.3 kg/m^2^, and excess of weight of 26 kg. Stratified data by gender is shown in Table 1.

### Initial Comorbidities

**Fig. 1:**
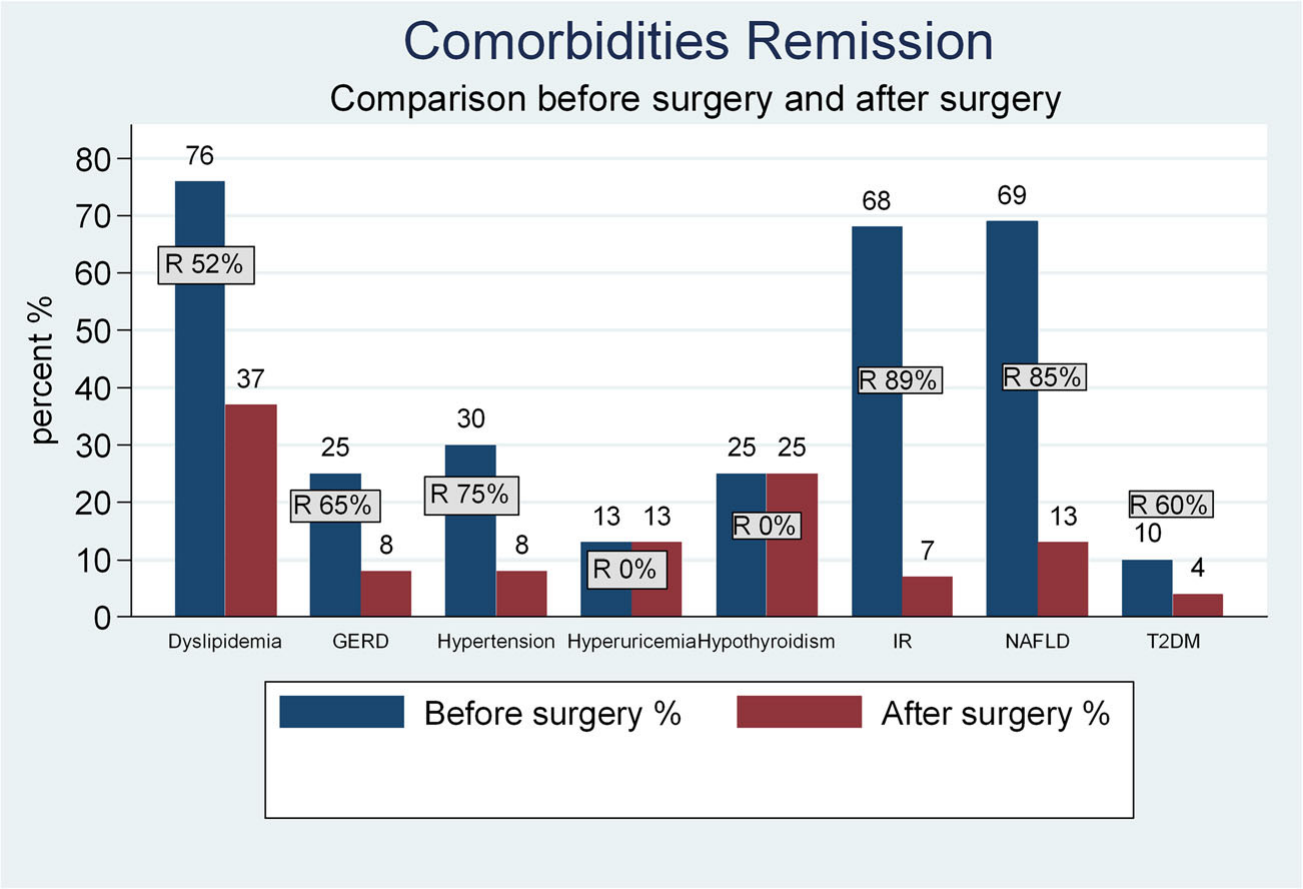
Comorbidity evolution before surgery and 3 years after surgery. Percent of patients and comorbidities before and after surgery. *NAFLD* non-alcoholic fatty liver disease, *IR* insulin resistance, *T2DM* type 2 diabetes mellitus, *GERD* gastroesophageal reflux, *R* remission

IR was 67.92%, T2DM 9.43%, dyslipidemia 76.19%, NAFLD 69.05%, HTN 30.19%, hyperuricemia 13.21%, and hypothyroidism 19.81%. A 25.43% of the patients had GERD symptoms before surgery, and 34% of them had a hiatal hernia associated that was repaired during surgery (Fig. 1).

The small number of diabetic patients included in our study attends to the fact that exclusion criteria established that diabetic patients with poor response to medical treatment were submitted to a RYGB.

### Postoperative Biometric Data

**Fig. 2:**
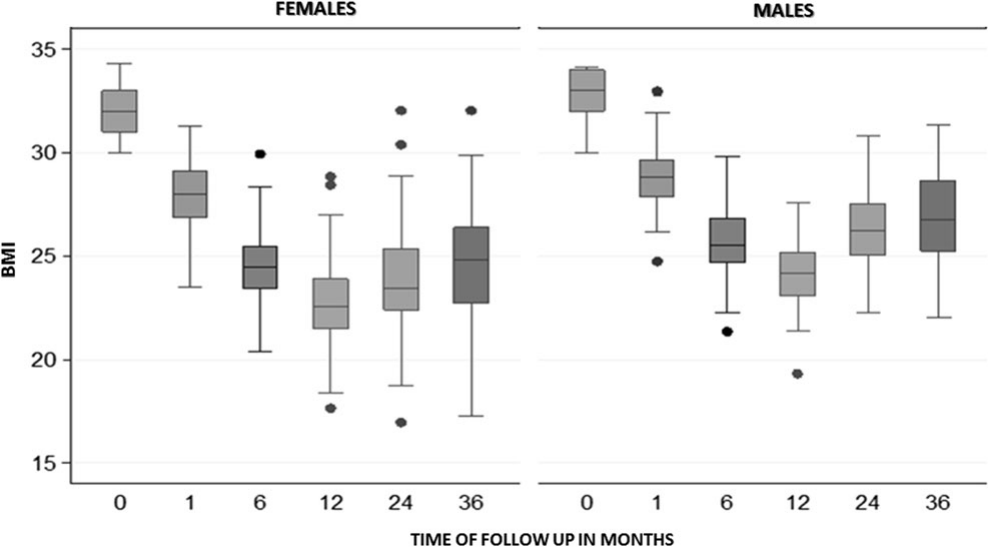
BMI Evolution during follow-up divided by gender. Evolution of body mass index (BMI) median and range by gender

Median postoperative BMI was 28.24, 24.78, 22.97, 24.14, and 25.1 kg/m^2^ at 1, 6, 12, 24, and 36 months, respectively, as we can see in Table 2. In Fig. 2, BMI evolution by gender is shown. Median %TWL was 12.9, 23.2, 28.2, 24.3, and 22.1% at 1, 6, 12, 24, and 36 months, respectively (*p* = 0.0001). Median %EWL was of 42.88, 77.44, 98.42, 83.2, and 75.79% at 1, 6, 12, 24, and 36 months, respectively. Evolution of BMI compared to initial values was statistically significant (*p* < 0.0001) (Table 2).

It is remarkable that only after 6 months of operation, all of the patients were no longer obese (BMI <30 kg/m^2^). Surgical weight loss success was achieved in 29% of the patients at the first month after surgery, and 94.6, 98.2, 88.3, and 84.3% at 6, 12, 24, and 36 months, respectively (Table 3).

### Comorbidity Remission

In Fig. 1, we compared comorbidity distribution before surgery and its remission afterward. Among patients with IR, 89.4% had normalized HOMA index with no medical treatment (*p* < 0.001). Those who used to take two or more drugs showed reduction to one drug in 67 and 33% had no need of medication anymore (*p* < 0.001). Those who used to take one drug had a complete remission in 90%. T2DM complete remission was observed in 60% of the diabetic patients, who presented normal measures of glucose metabolism and no longer medication requirement (*p* < 0.001). The remaining 40% of diabetic patients improved by achieving better glycemic controls with an A1C <7% and medication reduction in a 64.7% of the cases.

Dyslipidemia was remitted in 52% of the dyslipidemic patients (*p* < 0.001). Patients with NAFLD before surgery got normal or improved sonography in 84.6% (*p* < 0.001), evidenced between 6 and 12 months after surgery. One patient developed a liver hemosiderosis during the first year after surgery and another evolved to cirrhosis, 2 years later. Of the patients with HTN, 75% left their medication (*p* < 0.001), with normal blood pressure controls, and 25% had medication reduction, except for one patient who had a renal chronic disease before surgery and needed kidney transplantation afterward. There was no change in hypothyroidism and hyperuricemia.

Regarding GERD and/or hiatal hernia, 25.4% of the patients included in the study presented GERD symptoms but only 13.9% of them had endoscopic evidence of esophagitis (7.7% grade I esophagitis and 6.2% grade B esophagitis). Patients with grade C, grade D esophagitis, or Barret were excluded and a RYGB was performed. Hiatal hernia was found and repaired during surgery in 33.9%. After surgery, 64,6% of the symptomatic patients had an improvement of their symptoms (*p* < 0.001), of those, 25% had fewer symptoms but had to keep with proton pump inhibitors (PPI) treatment occasionally. A 20% had no change in their symptoms and 13% got worse with evidence of hiatal hernia or esophagitis progression. Two patients evolved to Barrett. De novo GERD was found in 2.4% (five patients).

### Surgical Data

Surgical time was 86.9 min (40–120). In eight of them, another abdominal simultaneous procedure was performed. Six of them were cholecystectomies because of cholelithiasis and two incisional hernia repairment. There were six cases of morbidity (2.4%) (Table 4). We had five early major complications: hemoperitoneum in two patients (0.8%), both of them had a good response to conservative medical treatment, one of them required a blood transfusion, but none of them required reoperation. One patient suffered abdominal pain 60 days after surgery, portal vein thrombosis was diagnosed, and it responded to anticoagulant treatment. One patient who had a cholecystectomy associated to his LSG presented a biliary peritonitis because of a Lushka leak. It was diagnosed in the first 24 hr and required reoperation to clip the Lushka duct. One patient had an acute umbilical trocar site hernia that required surgery to repair the hernia, with no intestinal compromise and good evolution afterward. There were two reoperations (0.8%), no leaks, and no mortality in this series. Only one of the excluded patients had a leak with good response to endoscopic treatment with an over the scope clip and a self-expanding stent but did not attend to further controls after 8 months.

## Discussion

Obesity is a disease that involves other severe conditions such as type 2 diabetes, arterial hypertension, sleep apnea, orthopedic diseases, and non-alcoholic fatty liver disease, among others, and all this situation interferes with work, family, and lifestyle [5–8].

Actually accepted indications for bariatric surgery are based on a National Institute of Health (NIH) consensus guidelines published in 1991. In this consensus, they indicate surgery for people with BMI over 40 and people with a BMI of 35 or more with at least two associated comorbidities [11].

However, surgery evolved in the last 20 years and bariatric procedures risks have decreased as surgical techniques were improved and surgeon’s experience grew [24, 25].

LSG is a technique that has been spread over the last 10 years, and in fact, it is not even mentioned in the 1991 NIH consensus guidelines. It has demonstrated to be safe and effective treating obesity and related diseases. It can be performed as a primary weight loss procedure or as an initial stage of a Biliopancreatic Diversion with Duodenal Switch (BPD-DS) [24–26].

It is also proved that LSG is more cost-effective than other bariatric procedures and also than treating comorbidities and complications in non-operated patients [26, 27].

Our findings agree with worldwide experience of LSG safety. Global morbidity in our series is 2.4% with no mortality and no severe complications as only two patients needed a reoperation among the considerable amount of 252 individuals included.

Safety, costs, and effectiveness are many of the reasons why LSG nowadays represents almost half of all the bariatric procedures performed in the USA, beating RYGB for the first time in history [28].

According to this, the American Society of Bariatric and Metabolic Surgery (ASMBS) position statement and guidelines agreed on the fact that people with class I obesity suffers from a physical, psychological, and social health burden and non-surgical therapies have sustained success only in a small part of them [13]. This group of patients has an increased risk of comorbidities, morbidity, and mortality independently of BMI [15]. Also, the Swedish Obese Subjects (SOS) study demonstrated that CVDs, coronary heart disease (CHD), cancer, and all-cause of mortality improve after surgery compared to those patients with medical treatment [29, 30]. Despite having the major number of patients and follow-up in the existing literature, its conclusions are still controversial as it is a non-randomized trial [30].

In our investigation, we observed that all of our patients with BMI below 35 suffered from different comorbidities such as insulin resistance, T2DM, dyslipidemia, NAFLD, HTN, GERD, hyperuricemia, and hypothyroidism. All of them had made hard efforts with previous medical treatments without sustained success, and surgery gave them the possibility to reach a healthy weight with resolution or remarkable improvement of their comorbidities with statistically significant outcomes.

Diabetes Surgery Summit recommends surgery for diabetic patients with class 1 obesity only in cases where all medical and lifestyle interventions for type 2 diabetes have failed [31, 32]. However, on the basis of our results, we think that this statement excludes patients with IR or recently diagnosed T2DM, who presented a remarkable remission rate in our series and could be prevented from reaching an advanced stage of their disease [33, 34].

Patients with GERD symptoms submitted to LSG presented an acceptable remission rate, and de novo GERD was low (2.4%) compared to other publications. This is probably because of the detailed and aggressive hiatus inspection during surgery and repair of all the hiatal hernias detected [35].

Analyzing weight loss, we found that it was astounding that only 1 month after surgery, all of the patients were out of obesity range with a %TWL of 12.9%. This may be related to the initial BMI that allows reaching a healthy weight earlier [36]. Also, %TWL and %EWL were achieved at the first postoperative year, even if it declined along the second and third year; a successful weight loss was maintained in a considerable amount of patients during the second and third year after surgery. Analyzing these outcomes, we agree with Ji Yeon Park and Yong Jin Kim when they describe that patients with BMI between 30 and 35 have better EWL and reach a lower BMI than patients with BMI over 35 at baseline [37].

### Strengths and Weaknesses

It is an important strength that a large number of patients were included in this study. As a high-volume center, our experience allows us to arrive at interesting conclusions.

On the other hand, being a retrospective study is an important weakness. Because of this, we had to deal with lack of information and insufficient follow-up, which made us exclude many cases.

Hopefully, further studies would generate the necessary evidence to allow a better patient selection for LSG based on their associated conditions and individual considerations instead of BMI.

## Conclusions

Class I obesity is accompanied by many comorbidities that treated in the early stages could avoid becoming more severe. Our study showed that LSG is a procedure that allows patients to reach a successful weight loss with considerable comorbidity remission and a low rate of complications.

## Appendix

**Table 1:** Patient demographics

	Females	Males	Total
No. (%) of patients	188 (75%)	64 (25%)	252 (100%)
Age (years)			
Median (IQR)	38 (17–67)	41 (15–70)	39 (15–70)
Initial weight (kg)			
Median (IQR)	84 (68–105)	101.85 (81.2–115)	86 (68–115)
Initial BMI (kg/m^2^)			
Median (IQR)	32 (30–34.3)	33 (30–34.1)	32.3 (30–34.3)
Excess weight (kg)			
Median (IQR)	27 (17.5–34)	25.6 (16.5–34)	26 (16.5–34)

*BMI* body mass index, *IQR* interquartile range

**Table 2:** BMI, %TWL, and %EWL evolution over a 3-year follow-up period after surgery

	1 month	6 months	12 months	24 months	36 months	*p*
BMI (kg/m^2^), median (IQR)	28.24 (23.5–32.9)	24.78* (20.4–29.9)	22.97* (17.6–28.8)	24.14* (16.9–32.0)	25.10* (17.3–32.0)	<0.0001
%EWL, median (IQR)	42.88 (14.8–96.8)	77.43* (30.5–152.4)	98.42* (29.4–177.8)	83.33* (13.7–169.4)	75.79* (12.5–163.6)	<0.0001
%TWL, median (IQR)	12.9 (4.3–20.5)	23.1* (9.3–38.0)	28.2* (9.8–46.4)	24.3* (3.7–45.4)	22.1* (4.1–43.4	<0.0001
Follow-up no. (%)	209 (82.6%)	223 (82.1%)	220 (87%)	128 (50.6%)	111 (43.9%)	

Continuous non-parametric variables are summarized in median and IQR

*BMI* body mass index, *%EWL* excess of weight loss percentage, *IQR* interquartile range

**p* < 0.0001 comparing %EWL evolution with 1° month

**Table 3:** Percentage of patients who achieved successful weight loss

	1 month	6 months	12 months	24 months	36 months
% of %TWL >20%	76	98	98	95	94
% of %EWL >50%	29	94.6	98.2	88.3	84.3

*%TWL* total weight loss percentage, *%EWL* excess of weight loss percentage

**Table 4:** Morbidity

Complication	Number	Percent
Hemoperitoneum	2	0.8
Portal vein thrombosis	1	0.4
Bile peritonitis	1^a^	0.4
Acute trocar site hernia	1^a^	0.4
Pleural effusion	1	0.4
Total	6	2.4

Morbidity, number of patients (no.), and percentages (%)

^a^Patients who required reoperation
